# LncRNA BRE-AS1 interacts with miR-145-5p to regulate cancer cell proliferation and apoptosis in prostate carcinoma and has early diagnostic values

**DOI:** 10.1042/BSR20182097

**Published:** 2019-03-29

**Authors:** Zhongjun Chen, Ming Zhen, Jiajie Zhou

**Affiliations:** Department of Urology, Jingzhou Central Hospital, The Second Clinical Medical College, Yangtze University, Jingzhou City 434020, Hubei Province, P.R. China

**Keywords:** apoptosis, lncRNA BRE-AS1, miR-145-5p, prostate carcinoma, proliferation

## Abstract

Long non-coding RNA (LncRNA) BRE-AS1 has recently proven to be a tumor suppressor in lung cancer. The present study aimed to investigate the involvement of lncRNA BRE-AS1 in prostate carcinoma (PC). In the present study we found that plasma BRE-AS1 and miR-145-5p were both down-regulated in PC patients than in healthy controls. Down-regulation of BRE-AS1 and miR-145-5p effectively distinguished early-stage PC patients from healthy controls. A significant and positive correlation between BRE-AS1 and miR-145–5p was only found in PC patients. BRE-AS1 overexpression mediated miR-145-5p up-regulation in PC cells, while miR-145-5p overexpression did not significantly affect BRE-AS1. Overexpression of BRE-AS1 and miR-145-5p led to inhibited proliferation and promoted apoptosis of PC cells. miR-145-5p inhibitor attenuated the effects of BRE-AS1 overexpression on cancer cell behaviors. Therefore, lncRNA BRE-AS1 may regulate cancer cell proliferation and apoptosis in PC by interacting with miR-145-5p.

## Introduction

Prostate carcinoma (PC) as one of the most frequently diagnosed cancers amongst males is a major cause of cancer-related deaths [1]. Risk of PC increases with ageing [2], besides that, fat intake, vitamin D deficiency, oxidative stress, hormones, race, and many other internal and external factors have also been proven to be closely correlated with the occurrence of PC [3]. However, the molecular pathogenesis of PC is still largely unknown, leading to failures in clinical treatment [4]. Therefore, in-depth investigations of molecular pathways involved in the development and progression of PC may guide the treatment.

The human genome transcribes both protein-coding mRNAs and non-coding RNAs (ncRNAs) [5]. Different from the functions of mRNAs as template of protein synthesis, ncRNAs participate both in physiological and pathological processes directly in the form of RNA [6,7]. According to the length and functionality, ncRNAs are divided into different subgroups, such as long ncRNAs (lncRNAs) and microRNAs (miRNAs), which are essential players in cancer biology [8,9]. LncRNA BRE-AS1 has recently proven as a tumor suppressor in lung cancer [10], while its role in other human diseases is unknown. It has been reported that miR-145-5p inhibits PC [11]. In the present study, we showed that lncRNA BRE-AS1 regulated cancer cell proliferation and apoptosis in PC possibly by interacting with miR-145-5p.

## Materials and methods

### Patients, specimens, and cell lines

A total of 64 patients with PC and 55 healthy volunteers who were admitted by Jingzhou Central Hospital from May 2015 to May 2018 were enrolled to serve as research subjects. Blood was extracted from each participant before breakfast in the morning at day 1 after admission. Cancer tissues as well as adjacent non-cancer tissues (within 2 cm around tumors) were collected from each patient before therapies. Patients’ inclusion criteria: (i) patients diagnosed by pathological biopsies; (ii) patients with normal functions of other major organs; (iii) patients who were diagnosed for the first time; (iv) patients with complete medical record. Patients’ exclusion criteria: (i) patients who were combined with multiple diseases and (ii) patients who were treated within 3 months before admission. The 55 healthy volunteers were selected to match distributions of patient group. Age of patient group ranged from 39 to 74 years and a mean age of 54.3 ± 5.6 years. According to AJCC stage, there were 14 cases at stage I, 16 cases at stage II, 18 cases at stage III, and 16 cases at stage IV. Age of control group ranged from 37 to 75 years and a mean age of 55.2 ± 6.1 years. All participants signed informed consent before admission. The present study passed the review of Ethics Committee of Jingzhou Central Hospital.

All *in vitro* cell experiments in the present study were performed using PC cell line 22Rv1. Cells of this cell line were purchased from American Type Culture Collection (ATCC, Manassas, VA, U.S.A.). DMEM containing 10% FBS (ATCC 30-2020) was used to cultivate the cells at 37°C in a 5% CO_2_ incubator.

### Real-time quantitative PCR

To detect the expression of lncRNA BRE-AS1, total RNAs were extracted using TRIzol reagent (Invitrogen, U.S.A.), reverse transcription was performed using Applied Biosystems™ High-Capacity cDNA Reverse Transcription Kit, and PCR systems were prepared using SYBR® Green Real-Time PCR Master Mixes (Thermo Fisher Scientific, U.S.A.) with GAPDH asan endogenous control. Primer sequences were: 5′-CCGCGGTGCCTGACAGTTCC-3′ and 5′-TAGTCTCGGTGCACAGCCTC-3′ for lncRNA BRE-AS1; 5′-CTGACTTCAACAGCGACACC-3′ (forward) and 5′-TAGCCAAATTCGTTGTCATACC-3′ (reverse) for GAPDH. To detect the expression of miR-145-5p, miRNAs were extracted using mirVana miRNA Isolation Kit (Thermo Fisher Scientific), reverse transcription was performed using miScript II RT Kit (QIAGEN Online), and PCR systems were prepared using mirVana qRT-PCR miRNA Detection Kit (Thermo Fisher Scientific) with U6 as the endogenous control. Sequence of forward primer of miR-145–5p was 5′-TGCCTCCAACTGACTCCTAC-3′. miR-145–5p reverse primer and U6 primers were included in the kit. Expression of lncRNA BRE-AS1 was normalized to GAPDH, and expression of miR-145–5p was normalized to U6, using 2^−ΔΔ*C*^_T_ method.

### Cell culture and transfection

Vectors expressing BRE-AS1 and empty vectors were designed and constructed by Sangon (Shanghai, China). MISSION® microRNA Mimic hsa-miR-145-5P and Scrambled miRNA negative control (NC) were from Sigma–Aldrich. MISSION® Lenti microRNA Inhibitor Human hsa-miR-145-5p and miRNA Inhibitor Negative Control were also from Sigma–Aldrich. Cells of 22Rv1 cell line were cultivated overnight to reach 70–80% confluence. All cell transfections were performed using Lipofectamine 2000 reagent (Thermo Fisher Scientific, Inc.) with vectors at a dose of 10 nM and miRNAs at a dose of 40 nM. Cells treated with Lipofectamine 2000 reagent only were control cells. Cells transfected with empty vectors, Scrambled miRNA NC, or miRNA Inhibitor NC were NC cells.

### *In vitro* cell proliferation assay

Expression of BRE-AS1 and miR-145-5p was detected 24 h after transfection, cells were collected for *in vitro* cell proliferation assay using Cell Counting Kit-8 (Sigma–Aldrich). Only in cases of BRE-AS1 and miR-145-5p overexpression rates reached 200% and miR-145-5p knockdown rate reached 50%. In brief, cells were harvested to prepare single cell suspensions. Cell density was normalized to 3 × 10^4^ cells/ml. Cell suspensions were transferred to a 96-well plate with 0.1 ml cell suspension for each well. Cells were cultivated in an incubator (37°C, 5% CO_2_), followed by the addition of CCK-8 solution (10 μl) 24, 48, 72, and 96 h later. After that, cells were cultivated for additional 4 h, and OD values at 450 nm were measured.

### *In vitro* cell apoptosis assay

Expression of BRE-AS1 and miR-145-5p was detected 24 h after transfection, cells were collected for *in vitro* cell apoptosis assay using Cell Counting Kit-8 (Sigma–Aldrich). Only in cases of BRE-AS1 and miR-145-5p overexpression rates reached 200% and miR-145–5p knockdown rate reached 50%. In brief, cells were harvested to prepare cell suspensions using serum-free medium. Cell density was adjusted to 3 × 10^4^ cells/ml. Cell suspensions were transferred to a six-well plate, with 10 ml in each well. Cells were cultivated for 48 h. After that, cells were subjected to 0.25% trypsin digestion, followed by Annexin V-FITC (Dojindo, Japan) and propidium iodide (PI) staining. After that, flow cytometry was performed to detect apoptotic cells.

### Statistical analysis

All experiments in the present study were performed in triplicate manner, and data were recorded as mean ± S.D. Correlations between plasma levels of BRE-AS1 and miR-145-5p in both PC patients and healthy controls were analyzed by Pearson’s correlation coefficient. Diagnostic values of plasma BRE-AS1 and miR-145-5p for early-stage PC were analyzed by ROC curve analysis with early stage (stages I and II) PC as true positive cases and healthy controls as true negative cases. Comparisons between two groups were performed by unpaired *t* test. One-way ANOVA and LSD test was used for comparisons amongst multiple groups. Sixty-four patients with PC were divided into high (*n*=32) and low (*n*=32) BRE-AS1 groups, and the correlations between BRE-AS1 and patients’ clinicopathological data were analyzed by Chi-squared test. Differences with *P*<0.05 were statistically significant.

## Results

### Plasma BRE-AS1 and miR-145-5p were both down-regulated in PC

**Figure 1:**
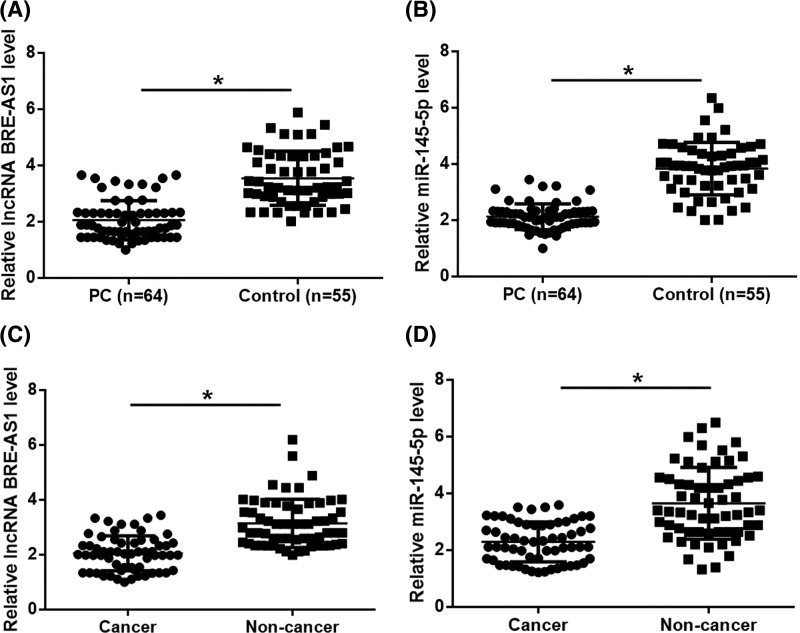
Plasma BRE-AS1 and miR-145-5p were both down-regulated in PC RT-qPCR results showed that, compared with healthy controls, plasma BRE-AS1 (**A**) and miR-145-5p (**B**) were both down-regulated in PC patients. It was also observed that expression levels of BRE-AS1 (**C**) and miR-145-5p (**D**) were both significantly lower in cancer tissues than in non-cancer tissues (*, *P*<0.05).

RT-qPCR was performed to detect the expression of plasma BRE-AS1 and miR-145–5p in both PC patients and healthy controls. Compared with healthy controls, plasma BRE-AS1 (Figure 1A) and miR-145-5p (Figure 1B) were both down-regulated in PC patients (*P*<0.05). RT-qPCR was ALSO performed to detect the expression of BRE-AS1 and miR-145-5p in cancer and non-cancer tissues of PC. It was observed that expression levels of BRE-AS1 (Figure 1C) and miR-145-5p (Figure 1D) were both significantly lower in cancer tissues than in non-cancer tissues (*P*<0.05). In addition, Chi-squared test showed that plasma BRE-AS1 and miR-145-5p were not significantly correlated with PC patients’ age, BMI, smoking and drinking habits (*P*>0.05), but was closely correlated with clinical stages (*P*=0.018 and 0.011, respectively). Similarly, expression levels of BRE-AS1 and miR-145-5p in cancer tissue were also not significantly correlated with PC patients’ age, BMI, smoking and drinking habits (*P*>0.05), but was closely correlated with clinical stages (*P*=0.006 and 0.005, respectively).

### Down-regulation of BRE-AS1 and miR-145-5p effectively distinguished early-stage PC patients from healthy controls

**Figure 2:**
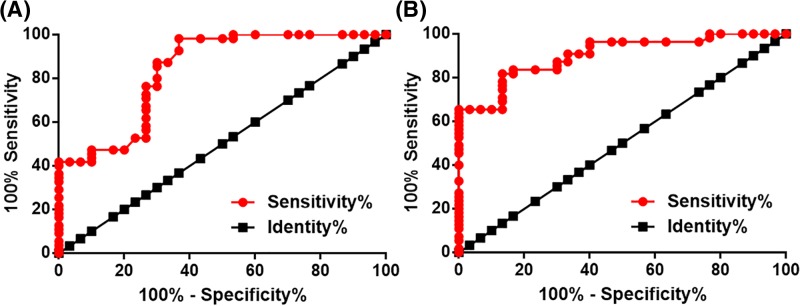
Down-regulation of BRE-AS1 and miR-145-5p effectively distinguished early-stage PC patients from healthy controls ROC curve analysis showed that down-regulation of BRE-AS1 (**A**) and miR-145-5p (**B**) effectively distinguished early-stage PC patients from healthy controls.

Our study included 14 PC patients at stage I and 16 at stage II, which were considered as early-stage PC patients. Diagnostic values of plasma BRE-AS1 and miR-145-5p for early-stage PC were analyzed by ROC curve analysis with early-stage PC as true positive cases and healthy controls as true negative cases. As shown in Figure 2, for plasma BRE-AS1, area under the curve was 0.84, with S.E.M. of 0.047 and 95% confidence interval of 0.75–0.93. For plasma miR-145-5p, area under the curve was 0.90, with S.E.M. of 0.032 and 95% confidence interval of 0.84–0.97.

### BRE-AS1 and miR-145-5p were positively correlated in PC patients

**Figure 3:**
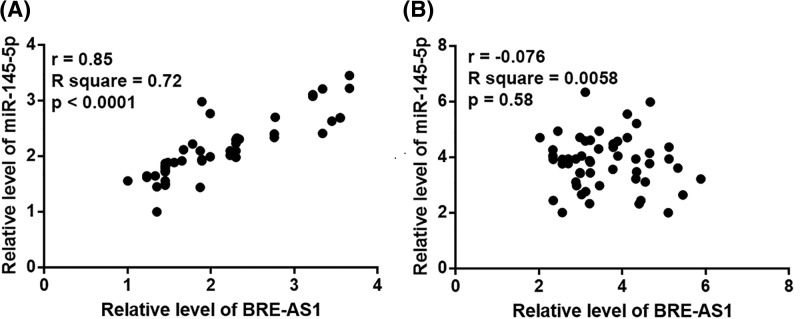
BRE-AS1 and miR-145-5p were positively correlated in PC patients Pearson’s correlation coefficient showed that BRE-AS1 and miR-145-5p were positively correlated in PC patients (**A**), but not in healthy controls (**B**).

Correlations between plasma levels of BRE-AS1 and miR-145-5p in both PC patients and healthy controls were analyzed by Pearson’s correlation coefficient. A significant and positive correlation between BRE-AS1 and miR-145-5p was only found in PC patients (r = 0.85, *P*<0.0001, Figure 3A). However, BRE-AS1 and miR-145-5p were not significantly correlated in healthy controls (r = −0.076, *P*=0.58, Figure 3B).

### BRE-AS1 is likely an upstream activator of miR-145-5p in PC

**Figure 4:**
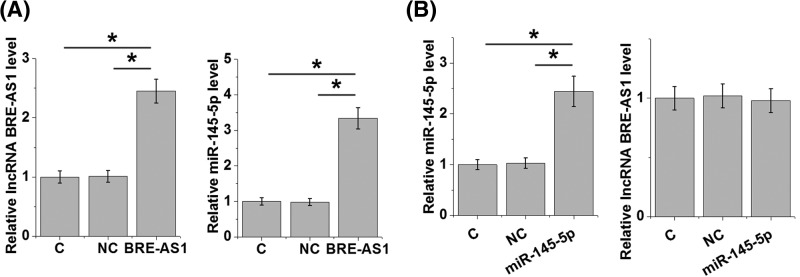
BRE-AS1 is likely an upstream activator of miR-145-5p in PC RT-qPCR results showed that BRE-AS1 overexpression mediated miR-145-5p up-regulation in cells of PC cell line 22Rv1 (**A**), while miR-145-5p overexpression did not significantly affect BRE-AS1 (**B**) (*, *P*<0.05).

Overexpression experiments were performed to further investigate the interactions between BRE-AS1 and miR-145-5p. RT-qPCR results showed that, compared with the control (C) and negative control (NC) cells. BRE-AS1 overexpression mediated miR-145-5p up-regulation in cell of PC cell line 22Rv1 (Figure 4A, *P*<0.05), while miR-145-5p overexpression did not significantly affect BRE-AS1 (Figure 4B).

### BRE-AS1 regulates the proliferation and apoptosis of PC cells through miR-145-5p

**Figure 5:**
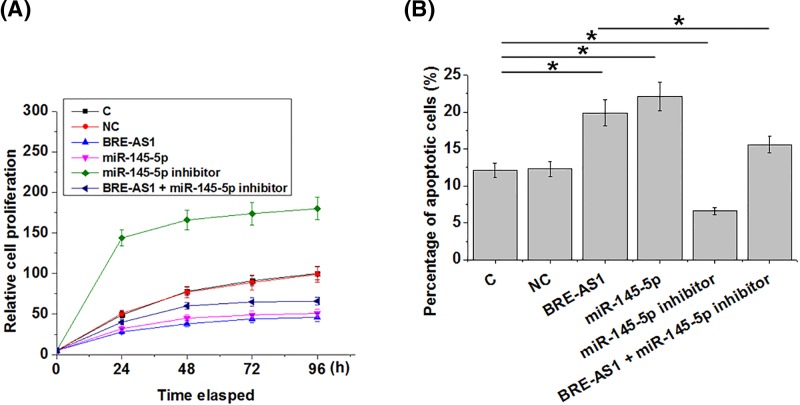
BRE-AS1 regulates the proliferation and apoptosis of PC cells through miR-145-5p Overexpression of BRE-AS1 and miR-145-5p led to inhibited proliferation (**A**) and promoted apoptosis (**B**) of PC cells. In addition, miR-145-5p inhibitor attenuated the effects of BRE-AS1 overexpression on cancer cell behaviors (*, *P*<0.05).

Compared and control (C) and NC cells, overexpression of BRE-AS1 and miR-145-5p led to inhibited proliferation (Figure 5A) and promoted apoptosis (Figure 5B) of PC cells (*P*<0.05). In addition, miR-145-5p inhibitor attenuated the effects of BRE-AS1 overexpression on cancer cell behaviors.

## Discussion

A recent study proved the role of lncRNA BRE-AS1 as a tumor suppressor in lung cancer [10]. Based on our knowledge, the functionality of lncRNA BRE-AS1 in other human diseases is unknown. The key finding of the present study is that lncRNA BRE-AS1 is also likely a tumor suppressor in PC and the actions of lncRNA BRE-AS1 in PC are likely mediated by miR-145-5p.

In spite of efforts made on cancer treatment, survival of patients with metastatic PC is generally poor due to the lack of radical treatment strategy [12,13]. Therefore, early diagnosis is still the key for the survival of PC patients. LncRNA BRE-AS1 was down-regulated in lung cancer [10]. In the present study, we first reported the down-regulation of lncRNA BRE-AS1 in PC. In effect, down-regulation of lncRNA BRE-AS1 effectively distinguished early stage PC patients from healthy controls. Therefore, lncRNA BRE-AS1 may serve as a potential biomarker for the early diagnosis of PC.

LncRNA BRE-AS1 represses cancer cell growth and survival in lung cancer [10]. Consistently, our study proved that lncRNA BRE-AS1 promoted apoptosis but inhibited apoptosis of cancer cells in PC, indicating the tumor suppressive roles of lncRNA BRE-AS1 in this disease. miR-145-5p is a well-characterized tumor suppression miRNA in different types of human cancers, and the silencing of miR-145-5p contributes to cancer development and progression [14,15]. A recent study reported that miR-145-5p inhibited the proliferation of PC [11]. Consistently, our study also observed the down-regulation of miR-145-5p in PC. In addition, our data also confirmed or proved the roles of miR-145-5p as an inhibitor of cancer cell proliferation and activator of cancer cell apoptosis in PC.

LncRNAs and miRNAs are two major subgroups of ncRNAs. A growing body of literature has shown that lncRNAs may interact with miRNAs to participate in physiological and/or pathological processes [16,17]. In the present study we showed that lncRNA BRE-AS1 was likely an upstream activator of miR-145-5p in PC, and the up-regulation of miR-145-5p by lncRNA BRE-AS1 participated in the regulation of proliferation and apoptosis of PC cells. Our findings provided new insights into the pathogenesis of PC. However, we speculated the interaction between miR-145-5p and lncRNA BRE-AS1 was indirect due to the observation that miR-145-5p and lncRNA BRE-AS1 were not significantly correlated in healthy controls.

Due to the limited information, the functionality of lncRNA BRE-AS1 in cancer biology is still largely unknown. However, it is known that miR-145-5p can participate in cancer biology through the interactions with multiple downstream oncogenic or tumor suppressive signaling pathways, such as transforming growth factor-β signaling [18], β-catenin signaling [19], and TAGLN2 [20]. Therefore, those downstream effectors of miR-145-5p may mediate the interaction between miR-145-5p and lncRNA BRE-AS1.

In conclusion, miR-145-5p and lncRNA BRE-AS1 were down-regulated in PC. LncRNA BRE-AS1 may regulate cancer cell proliferation and apoptosis in PC by up-regulating miR-145-5p.

## Informed consent

The study followed the tenets of the Declaration of Helsinki, and informed written consent was obtained from all patients and controls after we explained the nature and possible consequences of the study.

## Abbreviations

ATCC: American Type Culture Collection
BMI: Body mass index
DMEM: dulbecco’s modified eagle medium
GAPDH: glyceraldehyde-phosphate-dehydrogenase
lncRNA: long non-coding RNA
LSD: Least significant difference
NC: negative control
ncRNA: non-coding RNA
OD: optical density
PC: prostate carcinoma
ROC: receiver operating characteristic
TAGLN2: Transgelin-2
